# Editorial for “Gut Microbiota, Diet, and Gastrointestinal Cancer”

**DOI:** 10.3390/microorganisms14030716

**Published:** 2026-03-23

**Authors:** Marco Cintoni, Flavio De Maio

**Affiliations:** 1 UOC di Nutrizione Clinica, Dipartimento di Scienze Mediche e Chirurgiche, Fondazione Policlinico Universitario Agostino Gemelli IRCCS, Largo A. Gemelli 8, 00168 Rome, Italy; 2 Centro di Ricerca e Formazione in Nutrizione Umana, Università Cattolica del Sacro Cuore, 00168 Rome, Italy; 3 Department of Laboratory and Hematological Sciences, Fondazione Policlinico Universitario Agostino Gemelli IRCCS, Largo A. Gemelli 8, 00168 Rome, Italy; flavio.demaio@policlinicogemelli.it

## 1. Introduction

Gastrointestinal (GI) cancers remain a leading cause of morbidity and mortality globally, and their etiology is recognized as a multifactorial process driven by the interplay of genetic susceptibility, environmental exposures, and lifestyle-related factors [1]. In recent years, the gut microbiota (GM) has emerged as a key modulator within this complex landscape [2]. The bidirectional relationship between the host and its microbial communities influences not only local carcinogenic processes—through inflammation, immune dysregulation, and microbial metabolite production—but also systemic immune responses and the effectiveness of anticancer therapies [3].

In this Special Issue, “Gut Microbiota, Diet, and Gastrointestinal Cancer,” we compile nine articles, ranging from original research and pilot studies to comprehensive reviews, that collectively enhance our knowledge of how the microbiome serves as a diagnostic biomarker, a mechanistic driver of disease, and a promising therapeutic target.

## 2. The Microbiota as a Diagnostic and Prognostic Tool

Early diagnosis is crucial for increasing survival rates in gastrointestinal cancers [4]. Two studies in this collection examine non-invasive microbial biomarkers for colorectal cancer (CRC). Negrut et al. conducted a systematic review on the potential of the oral microbiome as a diagnostic tool, highlighting how some periodontopathogens, especially *Fusobacterium nucleatum*, are linked to CRC development and can be detected in saliva, making them a promising, non-invasive biomarker for screening [5]. Complementing this, Giacconi et al. conducted a case–control study of systemic biomarkers, focusing on plasma bacterial DNA load in CRC patients compared with healthy controls. Their findings showed significantly elevated plasma bacterial DNA levels in CRC patients that correlated with tumor mass, suggesting that bacterial translocation into the bloodstream could serve as a valuable indicator for early detection and risk stratification [6]. Despite the rapid advances in microbiome research, achieving clinically reliable and widely applicable microbial biomarkers remains a distant goal. Although *Fusobacterium nucleatum* is frequently discussed as a key CRC-associated microorganism, its interpretation as a single, uniform biomarker remains challenging due to the substantial genomic complexity within the *F. nucleatum* group and the ongoing taxonomic reclassification, which has introduced ambiguity between *F. nucleatum sensu lato* and *F. nucleatum sensu stricto* [7]. Moreover, the identification of unequivocally valid and reproducible microbiota-based biomarkers is further limited by the lack of harmonized pre-analytical and analytical procedures across studies, including differences in sample collection, processing, sequencing methodologies, and bioinformatic workflows. This insufficient standardization, and the frequent absence of an upstream normalization step, represents a major barrier to reproducibility and clinical translation [8].

## 3. Mechanisms of Carcinogenesis and the Impact of Dysbiosis

Understanding how microbes influence carcinogenesis is essential for the development of effective and targeted interventions [9]. Profir et al. provided a broad overview of the relationship between gut dysbiosis and digestive cancers, including malignancies of the esophagus, stomach, liver, pancreas, and colon. In particular, their review highlighted how dysbiosis may promote carcinogenesis through persistent inflammatory signaling, the production of microbial toxins (e.g., colibactin), and the modulation of the tumor microenvironment. In addition, the authors discussed the therapeutic potential of microbiota-targeted approaches, including probiotics and fecal microbiota transplantation [10].

Focusing more specifically on CRC, Cintoni et al. reviewed the literature with particular attention to the interplay between diet, microbiota composition, and clinical outcomes. Their comprehensive analysis emphasized that Western dietary patterns (characterized by a high intake of red and processed meats and a low intake of dietary fiber) promote a pro-carcinogenic microbial profile. Moreover, the authors highlighted the clinically relevant role of the microbiota in CRC management, including its influence on post-surgical outcomes such as anastomotic leakage, as well as its capacity to modulate the efficacy and toxicity of chemotherapy, radiotherapy, and immunotherapy [11].

Moving beyond the colon, Tavano et al. addressed the ongoing challenge of defining a “normal” pancreatic microbiome in order to better characterize microbial alterations associated with pancreatic ductal adenocarcinoma (PDAC). By comparing PDAC tissue samples with normal pancreatic tissues obtained from healthy organ donors, the authors identified a distinct intratumoral microbial signature. Notably, they reported a depletion of potentially beneficial bacteria, including *Acinetobacter guillouiae* and *Jeotgalicoccus*, in tumor specimens, together with metabolic alterations related to fatty acid biosynthesis, thereby providing novel insights into the contributions of microbes to pancreatic carcinogenesis [12].

Although primarily focused on cancer, a deeper understanding of the gut–brain axis is also relevant for gastrointestinal health more broadly. In this context, Pastras et al. reviewed the role of enteric nervous system (ENS) cells in irritable bowel syndrome. Their work elucidated molecular pathways linking the ENS and the microbiota, including brain-derived neurotrophic factor and Toll-like receptor 4 signaling, offering a mechanistic perspective on how dysbiosis may influence gut function and visceral sensitivity. Importantly, several of these pathways overlap with inflammatory processes that are also implicated in tumorigenesis [13].

## 4. Modulation via Lifestyle: Diet and Exercise

The possibility of modulating the gut microbiota through lifestyle-based interventions represents an emerging frontier for both cancer prevention and therapeutic support [14]. Yo et al. investigated the impact of physical activity in a high-fat-diet-induced obese mouse model of CRC. Their study showed that treadmill exercise significantly reduced colonic polyp formation and favorably reshaped the mucosa-associated microbiota, increasing *Akkermansia* relative abundance. Even though this genus appears inversely associated with inflammation and metabolic dysfunction [15], intraspecies genetic diversity should be taken into account, especially when *Akkermansia*-based probiotics are used.

Dietary regimens also exert a significant influence on gut microbial composition and function. Jo et al. conducted a pilot study examining changes in the gut microbiome during Ramadan fasting. In contrast to the beneficial effects commonly attributed to therapeutic intermittent fasting, they reported a reduction in both short-chain fatty acids and the abundance of potentially beneficial bacterial taxa during the fasting period, reflecting dietary patterns adopted during the feeding window. Interestingly, microbial diversity increased after Ramadan, underscoring the complexity and context dependence of dietary interventions targeting the microbiome [16].

Finally, with regard to nutritional supplementation, Onodera et al. explored the mechanistic basis of prebiotic support using human milk oligosaccharides (HMOs). Although HMOs, especially 2′-fucosyllactose (2′-FL) and 3′-sialyllactose (3′-SL), have been studied mostly with respect to infant nutrition, their application in adults is emerging as a highly relevant nutritional strategy for gastrointestinal health and cancer prevention. Their experimental findings demonstrated a cross-feeding interaction whereby *Bifidobacterium bifidum* metabolizes HMOs and releases intermediary sugars that can subsequently sustain the growth of the butyrate-producing commensal *Faecalibacterium prausnitzii*. Given that butyrate is widely regarded as a key oncostatic microbial metabolite, these results suggest that specific HMOs—such as 2′-FL and 3′-SL—may hold potential as components of adult nutritional strategies aimed at enhancing colonization resistance and strengthening gut barrier integrity [17].

## 5. Conclusions

This Special Issue underscores that GM is not merely a bystander in gastrointestinal cancers but an active player that can be leveraged from prevention to diagnosis and treatment [18]. From the oral cavity to the pancreas and colon, distinct microbial signatures track with disease states [19]. The collected works emphasize that while dysbiosis promotes disease through inflammation and genotoxicity, restoring balance through diet, exercise, and targeted prebiotics offers a viable therapeutic avenue [20]. Future research must focus on standardizing sampling and bioinformatic methods—the “normalization” of patients—that, together with longitudinal studies, may reduce causality, in order to translate these findings into personalized microbiome-based approaches for cancer patients.
